# Integrating Visual Perception and Control Strategies in Custom Omnidirectional Mobile Robots

**DOI:** 10.3390/s26123918

**Published:** 2026-06-20

**Authors:** Radu-Laurențiu Roșca, Andrei-Iulian Iancu, Adrian Burlacu, Cătălin Dosoftei

**Affiliations:** Faculty of Automatic Control and Computer Engineering “Gheorghe Asachi” Technical University of Iasi, 700050 Iasi, Romania; andrei-iulian.iancu@academic.tuiasi.ro (A.-I.I.); adrian.burlacu@academic.tuiasi.ro (A.B.); constantin-catalin.dosoftei@academic.tuiasi.ro (C.D.)

**Keywords:** omnidirectional mobile robotics, visual sensing, visual feedback control, robot operating system

## Abstract

Autonomous mobile robots are used in optimizing warehouse logistics, yet achieving precise positioning during docking maneuvers and autonomous planning remains a technical challenge. This study presents a custom vision-based control system designed for an autonomous omnidirectional wheeled robot. The proposed methodology acquires visual feedback using a stereo camera integrated within the Robot Operating System framework. Two visual feedback control laws are formulated and rigorously evaluated: a Classic Position-Based Visual Servoing algorithm, which minimizes pose error using a quaternion-based approach, and a second solution that utilizes Dual Lie Algebra to compute the 3D visual sensor’s velocities, ensuring convergence towards the desired point-feature configuration. Experimental validation reveals that while both methods achieve docking, the dual pose-free approach enables more robust, effortless movement of the robot platform than Classic Position-Based Visual Servoing. Consequently, these findings indicate that integrating depth-based feature recovery with advanced algebraic strategies offers a stable control strategy for automated industrial scenarios.

## 1. Introduction

Intelligent robotic systems have emerged as indispensable components in navigating dynamically complex environments across diverse domains, including manufacturing, healthcare, and everyday applications. The rapid advancement of mobile robotics technology has been largely driven by the growing demands of the intelligent manufacturing and logistics sectors. In particular, autonomous mobile robots have become integral to modern smart factory operations, where they must execute a broad spectrum of locomotion tasks with precision and reliability. The inherently complex and variable nature of such operational environments requires that mobile robotic platforms exhibit a high degree of flexibility, adaptability, and operational safety. Conventional differential drive mobile robots, however, present significant limitations in this regard due to their non-holonomic kinematic constraints, which fundamentally restrict their maneuverability and spatial adaptability. To address these limitations and enable smooth, flexible material transport within confined industrial environments, an Omnidirectional Mobile Robot (OMR) incorporating four Mecanum wheels was developed. The proposed system integrates a multi-sensor configuration to enhance perceptual awareness and closed-loop control capabilities. Mecanum wheel-based drive mechanisms have gained considerable traction in the development of omnidirectional mobile platforms, which are increasingly being deployed in intelligent warehouse and automated logistics operations [1]. The concept of movements without limits, implemented through these special wheels, offers important advantages over non-holonomic mobile robots, such as Ackerman steering and differential drives, for moving in logistics areas. These environments are collaborative spaces among operators, shelves, conveyors, and forklifts. In these conditions, to improve operational efficiency, the introduction of additional autonomous mobile robots (AMRs) must be accompanied by greater maneuverability in this crowded space. Omnidirectional maneuverability is achieved by precisely controlling each wheel’s speed and direction independently.

Visual servoing constitutes an important pillar of modern robotic control theory. In recent years, the integration of advanced stereo vision systems has offered multiple advantages, including real-time depth estimation. The deployment of advanced, high-frame-rate stereo cameras has catalyzed highly robust implementations across Image-Based Visual Servoing (IBVS), Position-Based Visual Servoing (PBVS), and Hybrid Visual Servoing (HVS) architectures.

IBVS operates on the principle of computing the control error directly within the two-dimensional image plane of the optical sensor. IBVS maps the differential changes in the two-dimensional image features—such as extracted keypoints [2], lines [3] or image moments [4]—directly to the required joint velocities of the robotic platform [5]. The primary advantage of IBVS lies in its robustness to calibration errors and system noise. The controller continues to generate velocity commands until the features mathematically align in the image matrix [6]. However, stereo IBVS is constrained by mathematical and physical disadvantages. The transformation mapping between the two-dimensional image space and the three-dimensional task space is highly non-linear. This non-linearity can frequently lead to control singularities in which the interaction matrix loses full rank, resulting in unbounded velocity commands [7].

PBVS operates by reconstructing the explicit three-dimensional pose of the target object relative to the camera frame [8]. The feedback loop relies heavily on pose estimation algorithms to extract the precise three-dimensional translation vector and rotation matrix, which together represent the target’s pose within the Special Euclidean group $SE_{3}$. The control error function is defined as the geometric distance between the current pose estimate and the desired pose. Once the error is computed, the robotic kinematic model generates the necessary joint torques or velocities to drive the end-effector seamlessly to the target position. The dominant advantage of PBVS is the predictability of the camera and the robotic platform in three-dimensional space. Because the error is minimized directly in Cartesian coordinates, the robot naturally follows an optimal spatial trajectory [9]. Conversely, PBVS relies heavily on precise camera calibration and mathematically perfect three-dimensional feature reconstruction. If the stereo camera drifts over time, the system will converge to an incorrect physical location. Furthermore, pure PBVS provides no mechanism to keep the target within the camera’s optical field of view. As the robot moves, the target features may simply exit the sensor’s field of view [10].

Hybrid Visual Servoing (2.5D Visual Servoing) integrates the advantages of IBVS and PBVS while discarding their vulnerabilities. In 2.5D partitioned visual servoing, the fundamental control law is mathematically decoupled. The camera’s rotational velocity is estimated by evaluating the homography matrix to determine the partial camera displacement in three-dimensional space, closely resembling PBVS. Simultaneously, the translational velocity of the system is controlled directly in the two-dimensional image plane, mirroring IBVS [11]. Alternatively, switching-based Hybrid Visual Servoing architectures constantly monitor specific geometric or state variables, such as target depth and the condition of the Jacobian matrix. Based on these observations, a logic controller dynamically transitions the control command between a pure IBVS controller and a pure PBVS controller, depending on the spatial proximity to the target or the calculated risk of field-of-view loss [12].

This paper presents the design of an omnidirectional mobile platform and evaluates two control strategies within the Position-Based Visual Servoing (PBVS) framework. The experimental results demonstrate that the performance of Classical PBVS is significantly enhanced by using a dual-number-based formulation.

## 2. Custom OMR System Architecture

### Custom Design for an Autonomous Omnidirectional Wheeled Robot

Modern warehouse logistics demand precision and adaptability, particularly when handling heavy payloads in highly constrained environments. In this section, the focus will be on the design and implementation of a custom-built Omnidirectional Wheeled Mobile Robot engineered specifically for these challenges. By integrating a dual-stereo camera perception system, this project solves critical bottlenecks in autonomous docking, precise spatial positioning, and reliable package delivery. Supplementary video footage validating the docking and payload tests can be viewed at https://youtu.be/e9q2MC5HzEs (accessed on 1 April 2026).

From a hardware perspective, the Omnidirectional Mobile Autonomous Robot is a mechatronic assembly that focuses on the mechanical elements and associated drive structures, which, based on the energy provided by the power system (usually of an electrical nature), act in the workspace to perform various tasks with the ability to understand the environment through the perception system. The decision-making component is usually implemented on a multi-layered control architecture. The software component that integrates various working algorithms must meet several important objectives, as defined by [13]: programmability, reactivity, autonomy and adaptability, robustness, coherent behavior, evolution, and observability.

Managing the complexity associated with high-performance omnidirectional robotic platforms requires the adoption of a structured, layered control framework. Within this framework, authority flows exclusively from higher-level layers to lower-level ones, establishing a well-defined order of precedence among functional components. Beyond simplifying system management, this organization provides considerable design freedom, since changes to a single module have negligible effects on the rest of the architecture. Two principal paradigms exist for formulating the control problem of a mobile robot: one rooted in kinematics and one rooted in dynamics. The kinematic formulation structurally separates the problem into two nested control loops operating at distinct timescales, whereas the dynamic formulation consolidates all control objectives into a single unified loop that captures the full system dynamics. Despite the theoretical appeal of the latter, its practical implementation is unattractive due to the substantial computational burden it imposes, making real-time execution demanding and the associated mathematical complexity significant. The kinematic formulation, being analytically more tractable, permits formal guarantees of closed-loop stability [14]. From a practical standpoint, the dynamic behavior of the robot need not be explicitly modeled, provided that the drive actuators have sufficient response time to commanded inputs at rates far exceeding those required by the task. Under this assumption, actuator torque can be treated as instantaneously available on the timescale of the higher-level control loops, thereby justifying a control design founded exclusively on the kinematic description of the platform [15].

**Figure 1:**
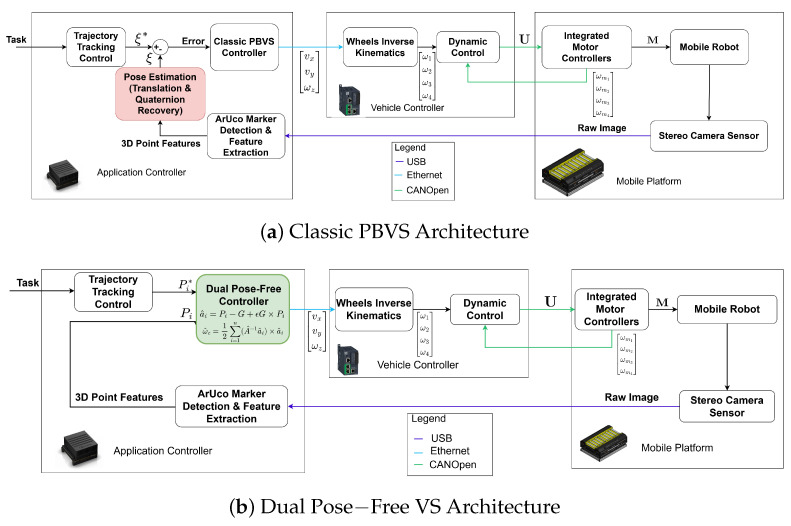
Architectural comparison between (**a**) Classic Position-Based Visual Servoing and (**b**) the proposed Dual Pose−Free Visual Servoing. The classic framework requires a computationally rigid intermediate step to estimate the full rigid-body pose, making the control loop highly vulnerable to visual occlusions. The proposed dual approach directly maps extracted 3D point features ($P_{i}$) into a dual−vector parameterization block, bypassing traditional intermediate rigid−body pose estimations.

As depicted in Figure 1, the proposed control structure is organized as a three-tier cascade, encompassing a trajectory generation loop at the outermost level, an intermediate kinematic loop, and an innermost dynamic loop. The stability of the composite system is ensured by maintaining a sufficient timescale across the three tiers, so that each inner loop operates on significantly faster dynamics than its enclosing outer loop [16]. The role of the vehicle controller is to aggregate motion commands from various sources, resolve conflicts using a defined priority scheme, and enforce kinematic feasibility by saturating both velocity and acceleration commands to keep the platform within its physical limits before dispatching the resulting signals to the actuators. Actuator velocity references are derived through the application of the inverse kinematic mapping of the OMR, which relates the local velocity vector comprising the longitudinal component $v_{x}$, the lateral component $v_{y}$, and the yaw rate $\omega_{z}$ to the individual angular velocities $\omega_{i}$ ($i=\bar{1,4}$) of the four wheels. This mapping accounts for the geometric parameters of the platform as introduced in [16], namely the wheel radius *R*, the lateral half-track $l_{x}$, and the longitudinal half-wheelbase $l_{y}$. The resulting inverse kinematic relationship, in matrix form, is given by Equation (1):

(1) $$\left[\begin{array}{c} \omega_{4}\\ \omega_{3}\\ \omega_{2}\\ \omega_{1} \end{array}\right]=\frac{1}{R}\left[\begin{array}{ccc} 1 & -1 & \left(l_{x}+l_{y}\right)\\ 1 & 1 & \left(l_{x}+l_{y}\right)\\ 1 & -1 & -(l_{x}+l_{y})\\ 1 & 1 & -(l_{x}+l_{y}) \end{array}\right]\left[\begin{array}{c} v_{x}\\ v_{y}\\ \omega_{z} \end{array}\right].$$

The vehicle controller uses the direct kinematic model (2) to determine the actual OMR’s relative speed to ground:

(2) $$\left[\begin{array}{c} v_{x}\\ v_{y}\\ \omega_{z} \end{array}\right]=\frac{R}{4}\left[\begin{array}{cccc} 1 & 1 & 1 & 1\\ -1 & 1 & -1 & 1\\ \frac{1}{l_{x}+l_{y}} & \frac{1}{l_{x}+l_{y}} & \frac{-1}{l_{x}+l_{y}} & \frac{-1}{l_{x}+l_{y}} \end{array}\right]\left[\begin{array}{c} \omega_{4}\\ \omega_{3}\\ \omega_{2}\\ \omega_{1} \end{array}\right].$$

Supplementary, as illustrated in Figure 1, the proposed system architecture relies on a highly distributed hardware topology. The perception cycle originates at the mobile platform, where the ZED 2 Stereo Camera acquires raw visual data. The image is transmitted to the Application Controller, where a dedicated ArUco detection node extracts the 3D point features ($P_{i}$) of the target. A fundamental difference between this proposed architecture and Classic PBVS lies in the way visual feedback is handled. Classic PBVS requires a fragile, computationally expensive intermediate step to extract the target’s exact rigid-body pose (translation and rotation) before computing a control error. The dual pose-free architecture fundamentally eliminates this intermediate bottleneck. Using Dual Lie Algebra, the controller parametrizes the raw 3D point measurements directly into dual vectors (${\hat{a}}_{i}$) and calculates the velocity field ($\hat{\omega_{c}}$). The control algorithm generates the platform velocity vector ${\left[v_{x},v_{y},\omega_{z}\right]}^{T}$. This high-level command is then given to the vehicle controller, who applies the inverse kinematics matrix to distribute specific angular velocities (${\left[\omega_{1},\omega_{2},\omega_{3},\omega_{4}\right]}^{T}$) to the integrated motor controllers.

The hardware architecture can be described by the simplified schematic in Figure 1, which illustrates the connectivity among low-level components required to drive the robot and to provide sensory feedback, as well as the network connections and communication protocols between high-level components. The system shown in this paper is composed of three computational units:

- **Vehicle Controller**: Implemented by an industrial Programmable Logic Controller (PLC) used for implementing the control logic for the movement of the robot based on the kinematic equations and the constraints imposed by the safety controller, and also managing the execution of the actuators. The reference velocity inputs can come either manually from a user using a joystick or using a numerical unit (in this case, the NVIDIA Jetson AGX Xavier) that communicates via a network connection with the PLC.
- **Safety Controller**: Its duty is to secure the safety of both the robot and the objects or people in its proximity, based on data acquired from an LiDAR system. The data represents distances to nearby obstacles, and the system uses this data to determine whether an object is in any of the user-defined regions around the robot, triggering control signals that tell the Schneider PLC to either slow down or stop the robot to avoid a collision.
- **Application Controller**: The most powerful processing unit in the system, which integrates and executes all of the software functionalities needed for local decision-making and data acquisition that require computationally heavy algorithms, which work with a big volume of data that needs to be acquired from network communication or the peripherals of the unit. The response of such algorithms needs to be fast to compensate for the network latency and to ensure the stability of the system and communicate anomalies, whether external, in the outside environment, or internal, in the system, so it can be as dynamic as possible, hence the need for computational power.

The software architecture is designed to prioritize modularity, scalability, and maintainability. Furthermore, it requires robust interfaces for efficient data acquisition, analysis, and debugging. To meet these functional requirements, the Robot Operating System (ROS) Noetic [17] framework was selected as the core development environment.

**Figure 2:**
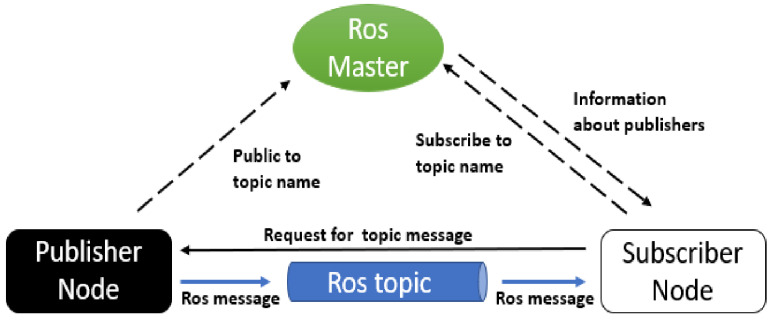
ROS architecture.

ROS operates in a simple, structured way and offers a highly versatile set of tools for robotics across most programming languages, for both development and visualization. The development tools help break complex functionality into nodes in a directed graph, making it easier to follow the system’s workflow and understand each individual subsystem. Any node can be a *publisher*, *subscriber*, or both (Figure 2). *Publishers* transmit data to the *subscribers* to listen through a *topic*. Abstractly speaking, a *topic* represents an edge between two nodes in the graph, where its purpose is to define the type of message that needs to be sent between two nodes, and which *publishers* communicate to which *subscribers*, where data is transmitted asynchronously.

The management of these functionalities is handled by the ROS Master, which adds nodes, ensures connectivity between them, whether they are on the same local network or not, and orders messages sent by *publishers* and in requests/responses.

## 3. Visual Perception and Control Strategy

The proposed control architecture for our custom Omnidirectional Mobile Robot prioritizes reliable visual perception for warehouse maneuvers. Using ArUco markers [18] and a dual-stereo camera integrated into the ROS-based Application Controller, the robot achieved robust docking and orientation. The proposed methods are Classic Pose-Based Visual Servoing and Dual Pose-Free Visual Servoing. In testing, the Dual Pose-Free Visual Servoing law outperformed the Classic Visual Servoing method, delivering a more stable and more accurate control response. In the YouTube video from Section Custom Design for an Autonomous Omnidirectional Wheeled Robot (Project Functionality Tests, at 1:00 min), the occupancy grid map of the operational environment is shown, which serves as the human–machine interface for the fleet management system. The map was constructed offline using simultaneous localization and mapping (SLAM) and is used at runtime for robot localization. The two orange rectangles represent the current poses of the two omnidirectional platforms (Rosy 1 and Rosy 2), with the green square marker indicating each robot’s heading. The current navigation goal vector is assigned by the task manager. To dispatch the leader to a docking location, an operator or high-level cloud task manager issues a target pose command specified as a 2D position and azimuth angle, which is forwarded to the leader’s navigation stack as a global goal. The robot then autonomously navigates to the vicinity of the target using odometric control mode. When the robot is close to the objective, the visual servoing controller is activated to execute the final precision docking maneuver. The follower robot receives no explicit goal from the task manager and remains in continuous visual servoing mode, maintaining its relative formation with the leader throughout the mission. This setup was used in order to validate the robustness and better performance of the Dual Pose-Free Visual Servoing controller within Section 4.

### 3.1. Visual Perception

The architecture design can be divided into two categories:

- Connections and communication between the hardware components.
- Frameworks and topology used for software functionalities.

Given the inherent complexity of the robotic platform, a distributed hardware architecture is adopted. This design ensures safe and reliable operation by distributing tasks across specialized components to prevent computational bottlenecks.

Stereo vision has proven to be effective in applications such as indoor navigation, obstacle detection and avoidance, or mapping buildings. In the field of autonomous robots, environmental dynamics are important factors in operational mode, response time, and safety measures. In this sense, a wide range of sensors is used to obtain information about the space in which the robot operates. Artificial vision systems are used in robot systems because they provide valuable information, such as depth, color, shape, and recognition of persons or objects, as well as, together with other sensors (barometer, magnetometer), positioning and orientation information.

Given these aspects, the stereo camera is a viable solution for this custom OMR project due to fast dynamics, the possibility of calculating the distance in real time, the ability to detect objects, shapes, and colors, along with the conditions of operation ensured by the working environment (optimum brightness, low presence of disturbances and exogenous elements). For this project, a ZED 2 camera manufactured by Stereolabs was used. It is a stereo camera used in other projects, such as quality control [19], visual SLAM [20,21], and underwater applications [22]. The camera can capture images at up to 2K resolution and is integrated with ROS, enabling the interconnection of the robot’s subsystems. This camera, along with the incorporated sensors (accelerometer, gyroscope, magnetometer), was used in project development for marker detection, positioning, and orientation.

The operational stability of the omnidirectional platform is highly dependent on the synchronized management of data across the distributed hardware architecture. To ensure safe and continuous trajectory execution, the data flow is strictly shared between the perception sensors, the Application Controller (NVIDIA Jetson AGX Xavier), and the vehicle controller (Schneider PLC). The perception cycle begins with the ZED 2 stereo camera, which is configured to capture high-definition RGB-D frames at 60 Hz. The raw optical and depth data are published into the ROS environment. A dedicated computer vision algorithm subscribes to this stream to detect fiducial markers. Upon successful detection, the node isolates the 3D point features ($P_{i}$) using the ArUco detect package, which implements the core ArUco algorithms explained in [23] and publishes the relative pose, fiducial area, and detection confidence within the ROS network.

While the camera hardware operates at 60 Hz, the visual feedback control node is implemented with a 10 Hz callback to avoid computational bottlenecks and network congestion. Within this 100 ms period, the Application Controller subscribes to the latest available asynchronous messages, parametrizes the 3D coordinates for the control law, and generates the final platform velocity command vector $u={\left[v_{x},v_{y},\omega_{z}\right]}^{T}$.

Once computed, the command vector is published to the network via the topic. The Schneider PLC (vehicle controller) acts as the subscriber for this high-level command. Operating on a significantly faster internal dynamic loop, the PLC receives the velocity command and instantly applies the inverse kinematics matrix to distribute the necessary angular velocities (${\left[\omega_{1},\omega_{2},\omega_{3},\omega_{4}\right]}^{T}$) to the four Mecanum wheel drives.

### 3.2. Visual Feedback Control

Visual feedback control integrates concepts from computer vision and control theory to guide robotic systems in performing various tasks. The goal of the control algorithm is to compute velocities to minimize the error between the desired position and the actual positions of the image features. Therefore, researchers have increasingly focused on visual servoing, developing innovative solutions for a range of sensor types and applications. To handle highly complex and unstructured environments, researchers are increasingly combining visual servoing with Deep Reinforcement Learning methods, as in [24,25]. Furthermore, cutting-edge predictive frameworks now incorporate visual servoing methods and visual feedback Control Barrier Functions (CBFs) to control robots in novel spatial environments and to guarantee real-time safety constraints for mobile robots navigating highly dynamic spaces [26,27].

#### 3.2.1. Classic PBVS

The Classic PBVS is designed to minimize the pose deviation between the current feature state and a defined reference state. For the rotational components, we utilize a quaternion-based representation, where a quaternion is expressed as $Q={\left[q_{0}\;q_{1}\;q_{2}\;q_{3}\right]}^{T}$, where $q_{0}$ is the scalar part and the units $q_{1}$ to $q_{3}$ form the vector part. Calculating the orientation error requires comparing the target quaternion $q_{ref}$ against the measured quaternion of the omnidirectional robot $q_{m}$. Adopting the methodology outlined by [28], the orientation error $q_{err}$ is obtained by multiplying the reference quaternion by the conjugate of the measured quaternion:

(3) $$q_{err}=q_{ref}*q_{m}^{*},$$

where $q_{ref}$ is the reference quaternion and $q_{m}^{*}$ is the conjugate of the estimated quaternion. The operator ∗ denotes the standard quaternion product (Hamilton product). For any two given quaternions $p={\left[p_{0},p_{v}\right]}^{T}$ and $q={\left[q_{0},q_{v}\right]}^{T}$, where $p_{0}$ and $q_{0}$ represent the scalar parts and $p_{v},q_{v}\in R^{3}$ represent the vector parts, the quaternion product is mathematically defined as

$$p*q=\left[\begin{array}{c} p_{0}q_{0}-p_{v}\cdot q_{v}\\ p_{0}q_{v}+q_{0}p_{v}+p_{v}\times q_{v} \end{array}\right].$$

The command that is transmitted to the motors is

(4) $$u=k_{p}\cdot \left[\delta_{X}\;\delta_{y}\;q_{1}err\right],$$

where $k_{p}$ is the gain and $\delta_{X}\;\delta_{y}$ are the position biases.

#### 3.2.2. Dual Pose-Free Visual Servoing

Pose-free visual servoing is a control framework that drives a robotic system directly from 3D point features, bypassing any intermediate recovery of the rigid object’s exact pose. The motion is parameterized through dual numbers and dual vectors, which together generate the orthogonal dual tensor group—a structure isomorphic to the special Euclidean group $SE_{3}$. A central contribution of this work is to adapt the pose-free scheme of [29] to the OMR architecture and to benchmark it against the classical PBVS.

The set of real dual numbers $\hat{R}=R+\varepsilon R$ is written as

(5) $$\hat{R}=\left\{\hat{a}=a+\varepsilon a_{0}|\;a,a_{0}\in R,{\;\varepsilon }^{2}=0,\varepsilon \neq 0\right\},$$

in which $a=Re(\hat{a})$ denotes the real part of $\hat{a}$ and $a_{0}=Du\left(\hat{a}\right)$ its dual part. Correspondingly, the set of dual vectors ${\hat{V}}_{3}=V_{3}+\varepsilon V_{3}$ reads

(6) $${\hat{V}}_{3}=\left\{\hat{a}=a+\varepsilon a_{0};a,a_{0}\in V_{3},{\;\varepsilon }^{2}=0,\varepsilon \neq 0\right\},$$

where $V_{3}$ is the three-dimensional linear space of free Euclidean vectors, and $a=Re(\hat{a})$ and $a_{0}=Du\left(\hat{a}\right)$ are, respectively, the real and dual parts of $\hat{a}$. Three operations are available on dual vectors: the scalar product (written $\hat{a}\cdot \hat{b}$), the cross-product (written $\hat{a}\times \hat{b}$), and the triple-scalar product (written $<\hat{a},\hat{b},\hat{c}>$).

Suppose a depth-capable visual sensor observes a rigid body in two configurations, a current and a desired one. The body is described by *n* point features, given in the current image as $p_{i}=\left(x_{i},y_{i}\right),i\in \left\{1,n\right\}$ and in the desired image as $p_{i}^{*}=\left(x_{i}^{*},y_{i}^{*}\right),i\in \left\{1,n\right\}$. From the sensor’s intrinsic parameters, together with the per-feature depth, the corresponding 3D positions $P_{i},P_{i}^{*},i\in \left\{1,n\right\}$ are reconstructed. The objective is to determine the 3D sensor velocities that steer the features toward the desired configuration. Denote the centroids of the 3D point sets ${\left\{P_{i}\right\}}_{i=\bar{1,n}}$ and ${\left\{P_{i}^{*}\right\}}_{i=\bar{1,n}}$ by

(7) $$G\left(t\right)=\frac{\sum_{i=1}^{n}P_{i}\left(t\right)}{n};G^{*}=\frac{\sum_{i=1}^{n}P_{i}^{*}}{n}.$$

The image error $p_{i}-p_{i}^{*}$ is back-projected into 3D using the intrinsic parameters and the depth measurements:

(8) $$E\left(t\right)=P_{i}\left(t\right)-P_{i}^{*},i=\bar{1,n}.$$

Differentiating with respect to time yields

(9) $${\dot{E}}_{i}\left(t\right)={\dot{P}}_{i}\left(t\right),i=\bar{1,n}.$$

Imposing an exponential decay of the error,

(10) $${\dot{E}}_{i}\left(t\right)=-\lambda E_{i}\left(t\right),i=\bar{1,n},$$

and combining Equation (9) with $v_{i}\left(t\right)={\dot{P}}_{i}\left(t\right)$, it follows that the desired behavior is obtained whenever the velocity field of the rigid-body features satisfies

(11) $$v_{i}\left(t\right)=-\lambda_{P}E_{i}\left(t\right),i=\bar{1,n}.$$

With these quantities, the following dual vectors are formed:

(12) $${\hat{a}}_{i}=P_{i}-G+\varepsilon G\times P_{i},$$

(13) $${\dot{\hat{a}}}_{i}=v_{i}-v_{G}+\varepsilon \left(v_{G}\times P_{i}+G\times v_{i}\right),$$

for $i=\bar{1,n}$, with $v_{G}=-\lambda_{G}\left(G-G^{*}\right)$.

The pose-free formulation of the visual feedback problem [10,29] collects the desired linear and angular sensor velocities $\left[v_{c}\;\;\omega_{c}\right]=\left[v_{x}\;v_{y}\;v_{z}\;\omega_{x}\;\omega_{y}\;\omega_{z}\right]$ into a single dual vector ${\hat{\omega }}_{c}=\omega_{c}+\varepsilon v_{c}$, which encodes the prescribed velocity field:

(14) $${\hat{\omega }}_{c}=\frac{1}{2}\sum_{i=1}^{n}\left({\hat{A}}^{-1}{\hat{a}}_{i}\right)\times {\hat{\dot{a}}}_{i},$$

with $\hat{A}={\hat{a}}_{1}⊗{\hat{a}}_{1}+...+{\hat{a}}_{n}⊗{\hat{a}}_{n}$. Equation (14) is straightforward to implement and to test. For the omnidirectional robot, the applied command reduces to

(15) $$u=[v_{x}\;v_{y}\;\omega_{z}].$$

The experimental results obtained on the omnidirectional platform are presented next.

## 4. Experimental Results

**Figure 3:**
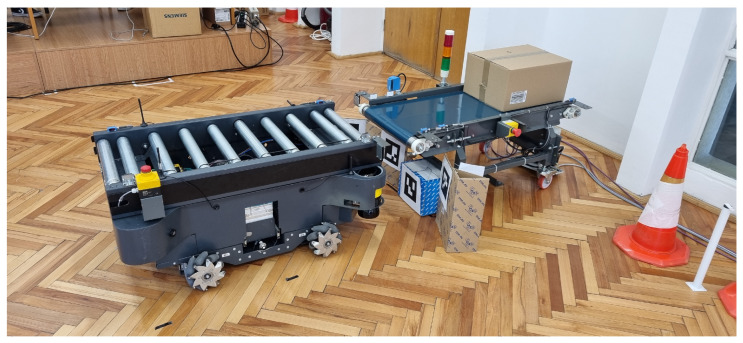
Marker-based docking.

The developed omnidirectional platform is illustrated in Figure 3. The same picture illustrates several markers considered for the project (16-bit binary matrix).

The camera captures HD images (720p resolution) at 60 Hz. In every frame, a computer vision algorithm detects ArUco markers. If one marker is detected, a ROS topic (e.g.,/fiducial_transforms) sends data through the system, including the relative pose of the marker with respect to the camera, fiducial area, and a confidence value for the detected object. Then, knowing the camera’s position relative to the robot’s center, the robot’s position is determined relative to the camera and the marker.

**Figure 4:**
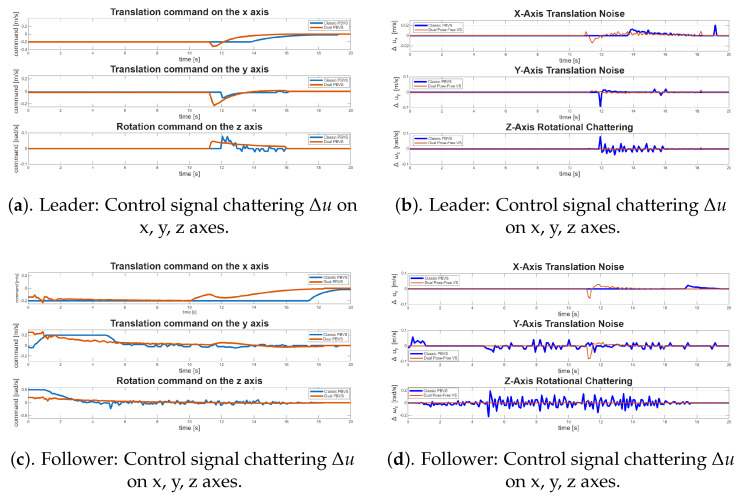
Comparison of Classic PBVS and Dual Pose−Free Visual Servoing velocity commands and control signal volatility for the leader (**top row**) and follower (**bottom row**) omnidirectional platforms.

To experimentally validate and compare the proposed control strategies, a dual-robot framework operating in a leader–follower configuration was established. It is important to note that the two platforms operate under fundamentally different control regimes throughout the mission. The leader robot is not continuously engaged in visual servoing; rather, it is dispatched by a task manager to a designated target location via a cloud-based command (Task and Trajectory Tracking Control from Figure 1). During the transit phase, the leader operates under waypoint-following control, and the visual servoing controller is activated only upon approaching the target, at which point the docking procedure is initiated. This behavior is reflected in the command profiles of Figure 4, where the leader’s visual servoing signals are constant during the initial transit and become active only after approximately 11 s. The follower robot operates in continuous closed-loop visual servoing mode throughout the entire duration, persistently tracking the leader as its dynamic visual reference. Since only the leader is assigned a physical destination by the task manager—as evidenced in the accompanying video—the follower’s sole objective is formation maintenance, making it permanently dependent on the quality and stability of its visual control law. In this way, the follower is exposed to a longer, continuous period of active visual servoing, making the robustness of the control law particularly critical to its smooth operation. To assess the performance of the visual servoing algorithms under different perceptual conditions, the experimental design incorporated two distinct fiducial marker arrangements: (i) a mono-marker configuration, featuring a single tag attached to both the docking station and the leader robot; and (ii) a multi-marker configuration, employing a group of four tags at each target location to describe the rigid body. The docking action starts by detecting the fiducial marker and extracting the translation vector and rotation quaternion. Using these data, the visual feedback algorithm minimizes the error between the robot’s desired docking position and its actual position. We have implemented a ROS node with a 10 Hz callback that processes data from the detection algorithm and computes a motor command, which is transmitted to the system via a dedicated topic (e.g., /cmd_vel).

These experiments aim to evaluate the differences between the two solutions described above: Classic and Dual Pose-Free VS. The goal is to compare linear and angular velocities and analyze the commands generated for the four mechanum wheels. The gains for the control laws were set to $k_{p}=0.3$ and $\lambda_{P}=\lambda_{G}=0.5$.

At HD resolution, the camera accurately detects a marker from 1.5–2 m up to 25 cm. At this distance, marker orientation is not precise, which is why the Dual Pose-Free VS method, which uses only marker positions, is preferred, thereby increasing the algorithm’s robustness.

The experimental results demonstrate a significant improvement in control signal integrity when utilizing the Dual Pose-Free VS formulation compared to the classic approach. Figure 4 presents the velocity command profiles and chattering for the dual-robot setup under both Classic PBVS and the proposed Dual Pose-Free Visual Servoing controller.

As depicted in Figure 4a, the Dual Pose-Free VS demonstrates a superior convergence trajectory. After 11 s, the dual method initiates a smooth, robust adjustment across all axes. The velocity commands approach zero asymptotically, indicating stable exponential error decay. In contrast, the Classic PBVS controller exhibits a delayed response followed by abrupt and aggressive step changes. This is most evident in the rotational Z-axis command, where the classic method forces the system into large-amplitude chattering throughout the task duration.

To rigorously quantify the impact of these algorithms, the derivatives of the control commands ($\Delta u_{x}$, $\Delta u_{y}$, $\Delta \omega_{z}$) were plotted to reveal high-frequency signals, indicating aggressive changes in acceleration that induce substantial mechanical stress on the actuators and gears. This phenomenon leads to increased energy consumption and potential hardware degradation. Figure 4b shows the advantage of the dual method, which mitigates the severe high-frequency oscillations seen within Classic PBVS. This chattering is caused by the intermediate pose estimation method, which is altered at close range. If the stereo camera is near the fiducial marker, minor depth ambiguities cause the pose recovery block to fluctuate rapidly, forcing the proportional controller to issue spiky commands. The Dual Pose-Free VS entirely solves this disadvantage. While there is a brief, bounded transient response as the maneuver initiates (t = 11 s), the derivative of the dual command quickly converges and remains near zero throughout the critical final docking phase.

The follower data corroborates the findings from the leader data, demonstrating the superiority of the Dual Pose-Free approach. As observed in the velocity command from Figure 4c, the Dual Pose-Free VS exhibits a much smoother and more anticipatory tracking behavior. On the X-axis translation, the dual method initiates a gradual, exponential deceleration phase significantly earlier. The Classic PBVS remains at −0.2 m/s for a longer duration, resulting in a late and aggressive kinematic correction. The Z-axis rotational command further highlights the dual method’s stability. The dual controller computes a low-amplitude, smooth trajectory. In comparison, the Classic PBVS initiates with an excessively high angular velocity (0.5 rad/s) and exhibits high-amplitude oscillations throughout the entire 20-s maneuver.

Figure 4d provides a better quantification of the high-frequency noise. The Classic PBVS controller exhibits severe chattering, particularly on the Y- and Z-axes. This phenomenon appears from the intermediate pose estimation algorithm, which induces noise into the controller. Classic PBVS struggles to maintain a consistent orientation while tracking the leader robot.

The Dual Pose-Free VS algorithm successfully filters out this instability. Because it directly parametrizes 3D feature points into a dual-vector velocity field, it is immune to pose estimation errors. While the dual method registers a single, brief transient spike on the X-axis and Y-axis at t = 11s, it immediately dampens the signal. Most importantly, the rotational chattering ($\Delta \omega_{z}$) is completely mitigated, remaining at a near-perfect zero for the entirety of the operation.

The seamless integration of robust visual perception with the continuous control strategies of a custom Omnidirectional Mobile platform is crucial for executing high-precision logistical tasks. Implementing a Pose-Free Visual Servoing architecture parameterized by dual vectors, the system achieves a high level of operational flexibility. Because the dual control law relies purely on the mathematical relationships and velocity fields of 3D point sets, the method can use a variety of point features, such as infrared point features, Artificial Fiducial Markers (e.g., ArUco, AprilTags) and Deep Learning-Based Semantic Keypoints (e.g., SuperPoint). This represents an advantage over Classic Position-Based Visual Servoing (PBVS). Classic PBVS strictly requires an a priori 3D geometric model of the target to explicitly compute a rigid-body pose estimation before it can generate any control velocities. This intermediate estimation step is computationally rigid; if the robot rotates and a feature leaves the field of view, traditional PBVS often fails. Conversely, the pose-free dual method entirely addresses this vulnerability by directly computing the required velocities from the visible 3D measurements. If occlusions occur during a maneuver, the dual controller simply reduces the contribution of the occluded feature while maintaining trajectory stability. In this way, the Dual Pose-Free VS represents a robust method for pure visual tracking, enabling the omnidirectional robot to dock seamlessly in dynamic, highly unstructured warehouses.

Consequently, the Dual Pose-Free VS method exhibits superior trajectory generation capabilities, characterized by more intuitive, kinematically consistent robot behavior. The dual approach initiates a gradual deceleration phase much earlier in the trajectory; this way, the robot converges with a fluid motion that is both safer and more predictable.

The real behavior of the visually controlled omnidirectional robot can be observed in these two videos: https://youtu.be/scYwB4Gz-58 (accessed on 1 April 2026) and https://youtu.be/4BAgJl4RAQA (accessed on 1 April 2026).

## 5. Conclusions

This research aims to address the precision-positioning bottlenecks inherent to autonomous, high-payload warehouse logistics. This paper presents a custom OMR architecture and contributes to the design of an integrating protocol of visual perception and control. This platform was explicitly chosen because traditional differential-drive systems lack the lateral maneuverability required for docking and heavy-payload positioning in constrained warehouses.

Robust visual perception underpins the autonomous navigation and maneuvering capabilities of our custom Omnidirectional Mobile Robot. The primary feedback loop is driven by a stereo camera system paired with an ArUco fiducial marker detection pipeline, fully integrated into the Robot Operating System framework on the main application controller. By leveraging dense depth data from the stereo camera, the system achieves continuous marker extraction and tracking, demonstrating high resilience against variable illumination and dynamic warehouse environments.

Experimental validation of the proposed control strategies—Classic Pose-Based Visual Servoing and Dual Pose-Free Visual Servoing—revealed significant performance differences. The Dual Pose-Free controller outperformed the classic baseline, demonstrating superior steady-state positioning accuracy and smoother trajectories. Consequently, the integration of this dual method markedly enhanced the system’s overall robustness and minimized operational failure rates under dynamic conditions.

Another contribution highlights how visual feedback control can be integrated into the OMR architecture while achieving autonomous behavior. The evaluation of Classical Position-Based Visual Servoing and an innovative pose-free approach to visual servoing provides a solid foundation for integrating visual control. While Classical PBVS is computationally rigid—relying heavily on explicit a priori 3D models and risking control stability if features leave the field of view—the Dual Pose-Free VS method proved fundamentally more robust. The dual approach bypassed fragile intermediate pose estimations entirely, offering a highly flexible and robust alternative. Optimal kinematic behavior can be achieved by systematically tuning the two exponential-decrease parameters ($\lambda_{P}$ and $\lambda_{G}$). By allowing the robot to maintain operational continuity even if markers were lost, provided at least three markers were visible, the system demonstrated high resilience. This fault-tolerant capability is particularly advantageous in warehouse environments, where fluctuating lighting conditions and physical obstructions often interfere with visual tracking.

To validate these theoretical advantages within a practical logistical paradigm, the control architecture was deployed on a custom-engineered Omnidirectional Mobile Robot. Empirical results substantiated the operational superiority of the dual method, demonstrating sustained trajectory stability and precise autonomous docking execution. Notably, the pose-free algorithm dynamically discarded unobserved visual features, thereby maintaining control-loop continuity without compromising asymptotic convergence. By decoupling and scaling the translational and rotational error-decay rates, the parameter-tuning framework effectively mitigated mechanical overshoot, thereby ensuring smooth, precise, and bounded velocity profiles essential for transporting heavy logistical payloads. Future work will focus on extending this framework to multi-robot cooperative logistics and on integrating dynamic obstacle-avoidance capabilities.

## Data Availability

The datasets presented in this article are not readily available because these data are part of a broader ongoing research project focused on long-term fleet management. Requests to access the datasets should be directed to [the corresponding author].
